# Time to speed up the diagnostic evaluation in clinically suspected rhinosinusitis patients: A debate on the conventional versus molecular workup to establish fungal infective etiology for prompt management

**DOI:** 10.18502/cmm.8.1.9207

**Published:** 2022-03

**Authors:** Uneza Husain, Ragini Tilak, Sushil K. Aggarwal, Ketan Priyadarshi, Neeraj Dhameja

**Affiliations:** 1 Department of Microbiology, Institute of Medical Sciences, Banaras Hindu University, Varanasi, Uttar Pradesh, India; 2 Department of Otorhinolaryngology, Institute of Medical Sciences, Banaras Hindu University, Varanasi, Uttar Pradesh, India; 3 Department of Pathology, Institute of Medical Sciences, Banaras Hindu University, Varanasi, Uttar Pradesh, India

**Keywords:** Clinically suspected rhinosinusitis, Fungal Rhinosinusitis, Laboratory diagnosis, Molecular detection, Nasal tissue

## Abstract

**Background and Purpose:**

Rhinosinusitis (RS) is a clinical and radiological diagnosis that rarely reaches a proper infective etiological diagnosis. The most dreaded fact about invasive fungal rhinosinusitis is its poor prognosis in immunocompromised patients with a 60-80% mortality rate. The present study highlights and compares the various diagnostic techniques to establish a fungal etiological diagnosis in clinically suspected cases of RS from nasal biopsy specimens, with the emphasis on the molecular diagnostic approach.

**Materials and Methods:**

This prospective study included a total of 34 clinically suspected cases of RS who had recently undergone functional endoscopic sinus surgery (FESS)/biopsy from nasal polyps. Various laboratory methods (microbiological and histopathological) were utilized, including direct microscopic examination of clinical samples and fungal culture isolation. The molecular detection method of polymerase chain reaction (PCR) from clinical samples was also explored simultaneously. Serum immunoglobulin-E (IgE) testing of patients was also performed.

**Results:**

Out of 34 clinically suspected RS cases, fungal etiology was established in a total of 18 cases, 17 of whom were culture-proven. A total of 15 and 14 culture-proven cases were also detected on direct microscopic examination by potassium hydroxide (KOH) mount and histopathological staining, respectively. One case was additionally identified by molecular method. *Aspergillus flavus* complex was the most common pathogen isolated in culture. Allergic fungal RS was the most common type, followed by acute and chronic invasive types among all fungal RS cases.

**Conclusion:**

Accurate and prompt etiological diagnosis of fungal RS is still lagging with fewer options for quick results. Although microscopy and culture isolation can’t be replaced, PCR is a sensitive and specific method that should be incorporated as a supplementary tool for the early diagnosis and management, considering the delayed growth of fungi.

## Introduction

Rhinosinusitis (RS) is inflammation of the lining of the nose and the sinuses surrounding the nose [ 1 ]. It affects nearly 31 million Americans and results in 18 to 22 million medical visits annually in the USA [ 2 ]. It should be noted that RS is divided into acute, sub-acute, and chronic forms, according to the duration of symptoms. In acute, subacute, and chronic RS, symptoms last for up to four weeks, between 4 and 12 weeks, and beyond 12 weeks, respectively [ 3 - 5 ]. The cases of fungal rhinosinusitis (FRS) are increasing day by day in India. Although there is still much confusion regarding the classification, presently, FRS is categorized into invasive and non-invasive diseases, based on histopathological evidence of tissue invasion by fungi [ 6 - 9 ]. The invasive diseases include 1) acute invasive (fulminant) FRS, 2) granulomatous invasive FRS, and 3) chronic invasive FRS. The non-invasive diseases include 1) saprophytic fungal infestation, 2) fungal ball, and 3) fungus-related eosinophilic RS that includes allergic fungal rhinosinusitis (AFRS) [ 9 ]. Acute invasive (fulminant) FRS is characterized by acute neutrophilic infiltration and vascular invasion of fungi, and patients with this devastating form usually have immunocompromised status. Moreover, this disease has a high mortality rate in case it is not recognized early and treated aggressively. Chronic invasive FRS progresses slowly with such features as low-grade inflammation, dense hyphae, and involvement of local structures. In the cases of granulomatous invasive FRS, non-caseating granulomas are typically present along with giant cells, sparse hyphae, and perivascular fibrosis. Saprophytic fungal infestation is characterized by sinonasal tract colonization by fungi usually following a traumatic event/ surgical procedure, and it causes inflamed and ulcerated/ crusted sinonasal mucosa without tissue invasion. Sinus mycetoma/ball is a sequestration of fungal hyphal elements within the sinus without any invasive/granulomatous changes [ 9 ]. AFRS is the commonest form of FRS [ 10 ]. The diagnostic criteria for AFRS vary among authors. As described by Bent and Kuhn, the positive fungal stain is one of the five major criteria for AFRS, other than type 1 hypersensitivity to fungi, nasal polyposis, radiographic finding, and eosinophilic mucin [ 11 ]. However, diagnosis is confusing since sparse fungal hyphae are difficult to demonstrate in allergic mucin. This has led to the description of two new entities similar to AFRS, including eosinophilic fungal rhinosinusitis (EFRS) and eosinophilic mucin rhinosinusitis (EMRS). EFRS is described as FRS having fungal hyphae embedded in eosinophilic mucin with or without evidence of type I hypersensitivity, while EMRS is characterized by the presence of eosinophilic mucin without fungal hyphae [ 12 , 13 ]. The most dreaded fact about FRS is its poor prognosis in immunocompromised patients with a 60-80% mortality rate [ 2 ]. The diagnosis of RS can be made by a combination of methods, such as clinical, imaging, microscopy/histopathology, culture, molecular, and immunological tests [ 14 ]. Surgery is performed in patients with a recurrent or persistent infection that is not resolved by drug therapy or in cases of extra nasal spread of infection, mucocele or pyocele, fungal sinusitis, or obstructive nasal polyposis [ 15 ]. The present study highlights the various diagnostic techniques for the detection of fungi in nasal specimens with special emphasis on the molecular approach, which is the need of the hour. The present study aimed to evaluate the importance of molecular assays, especially polymerase chain reaction (PCR), as an adjunct to existing direct microscopic examination of clinical specimen and fungal culture to improve and speed up the diagnosis of FRS.

## Materials and Methods

This prospective study was carried out in the Department of Microbiology in collaboration with the Department of Pathology and Otorhinolaryngology in a tertiary care hospital in eastern India for a total duration of one and a half years from September 2016 to April 2018. Clinically suspected inpatients of RS admitted to the Otorhinolaryngology department with at least two major or one major and two minor clinical criteria were included in the study as clinically suspected cases of RS. The used criteria included (1) Major criteria: Facial pain/fullness, nasal obstruction, postnasal discharge, hyposmia/anosmia, and fever (2) Minor criteria: Headache, halitosis, fatigue, dental pain, cough, ear pain/fullness [ 16 ]. All patients who were already started on antifungal therapy were excluded from the study. Patients were evaluated for cheesy materials coming from the nose or the presence of nasal polyps by otorhinolaryngology surgeons. Any relevant history of allergy, debilitating diseases, diabetes mellitus, or immune-deficiency disorders was documented. 

The clinical samples obtained from the clinically suspected RS cases included (1) Samples obtained from para-nasal sinuses by functional endoscopic sinus surgery (FESS) or tissue biopsy from nasal polyps; and (2) Samples from venous blood for serum IgE estimation. A portion of the surgically excised nasal tissue or FESS sample was sent to the mycology laboratory in a sterile container containing normal saline, and another part of the specimen was sent in a sterile container containing 10% formalin for histopathological examination (HPE).

Direct microscopic examination of the formalin-fixed clinical specimen was performed following histopathological staining using various stains (e.g., Hematoxylin and eosin [H & E] stain, Periodic acid–Schiff (PAS) stain, and Grocott-Gomori Methenamine Silver [GMS] stain). Specimen collected in sterile normal saline was subjected to direct microscopic examination using 20% potassium hydroxide (KOH) mount and calcofluor white staining, in addition to fungal culture isolation. Samples were inoculated in duplicate on culture media, such as Sabouraud’s Dextrose Agar (SDA), Potato Dextrose Agar (PDA), Sabouraud’s Dextrose Agar with chloramphenicol and gentamicin (SCA), and 5% sheep blood agar (BA) and incubated at 25oC and 37oC aerobically and checked daily for any growth. The direct microscopy result was informed to the treating surgeon to facilitate early treatment. All cases were predominantly classified into acute fulminant invasive FRS (AFIFRS), granulomatous invasive FRS (GIFRS), chronic invasive FRS (CIFRS), AFRS, and non-allergic FRS, based on (HPE) [ 6 - 9 , 11 ]. The culture plates were examined for growth after overnight incubation and then every day for seven days. If no growth was observed, the culture media were reviewed thrice a week in the second week and twice a week till four weeks before it was called negative for any fungal growth. 

Upon the observation of fungal growth in culture media plates, it was examined for its colony morphological characteristics, such as growth rate, presence of mycelium, color, obverse, and reverse of growth or any pigment production. Slide cultures using cornmeal agar were performed in duplicate and examined after three days and five days. The tease mount was prepared from colony growth (when mature) using lactophenol cotton blue (LPCB) stain and examined for further identification of the organism. Total serum IgE estimation was performed using serum samples from the patients. Serum IgE value ≥100 IU/ml in an adult patient was considered positive. Fungal culture was considered the gold standard test in the present study and the direct microscopic examination of clinical specimens as well as molecular assays were evaluated with respect to culture isolation. The statistical significance was calculated using the Chi-square test, and the statistical analysis of data was conducted using SPSS software (Version 25).

Fungal nucleic acid was extracted from the direct clinical samples, and nested PCR was performed using universal pan fungal primers targeting the internal transcribed spacer (ITS) region [ITS-1 and ITS-2] of the fungal genome. Extraction of fungal DNA from the clinical sample was performed by Qiagen nucleic acid extraction kit (Qiagen, India) as per the manufacturer's kit instructions using lyticase solution (10 U/ml lyticase with 28 mM ß-mercaptoethanol, 50 mM Tris and 10 mM EDTA) and proteinase K lysis buffer. The DNA content and purity of the extracted DNA eluted in TE buffer (at 260/280 ratio and 260/230 ratio) were estimated using a spectrophotometer (NanoDropTM). Afterward, the extracted DNA was stored at -20oC for further use. 

The primary PCR cycle was carried out using the following pan-fungal primers- (1). Forward primer (ITS-1): 5’-TCCGTAGGTGAACCTGCGG-3' (2) Reverse primer (ITS-4): 5’-TCCTCCGCTTATTGATATGC-3'. The nested PCR cycle was carried out using the following primer pairs - (1) Forward primer (ITS-1): 5’-TCCGTAGGTGAACCTGCGG-3' (2) Reverse primer (ITS-2): 5’-GCTGCGTTCTTCATCGATGC-3' with an expected size of 200-300 bp of nested cycle amplicon. A total of 25μl master mix for PCR was prepared using 2.5µl buffer (10X), 2µl dNTP mix (10mM), 0.33µl Taq polymerase (3U/µl), 1µl (10pmol) forward and reverse primers [GeNei, Merck, India] along with 5µl extracted DNA as a template for primary cycle and 1µl of first-round PCR amplicon for second round nested PCR. Using a thermal cycler (Bio-Rad, USA), the reaction mixture was subjected to 10 mins of initial denaturation at 95°C, followed by 40 cycles consisting of 45 secs of denaturation at 95°C, 45 secs of annealing at 48°C and 51°C, respectively, for the first and second-round PCR and 90 secs of extension at 72°C, followed by 10 mins of final extension at 72°C. The amplified PCR product (10μl) was analyzed using electrophoresis in a 2% agarose gel (HiMedia, RM 273, India) stained with ethidium bromide with Tris/Borate/EDTA (TBE) buffer.

The PCR amplification of the human β-globin gene sequence was employed as an internal control to assess the extraction of adequate amplifiable DNA and the absence of PCR inhibitory substances in the extracted DNA. The DNA extraction and PCR protocol were optimized using various fungal reference strains. 

DNA sequence analysis was carried out for the nested PCR amplicons obtained from clinical samples, which were both culture and PCR positive for standardization and validation of PCR protocol and culture-negative PCR positive sample. Sequence similarity was assessed through searching for homology with GenBank sequences using Nucleotide Basic Local Alignment Search Tool (BLAST) software from the National Center for Biotechnology Information (NCBI).

## Results

**Figure 1:**
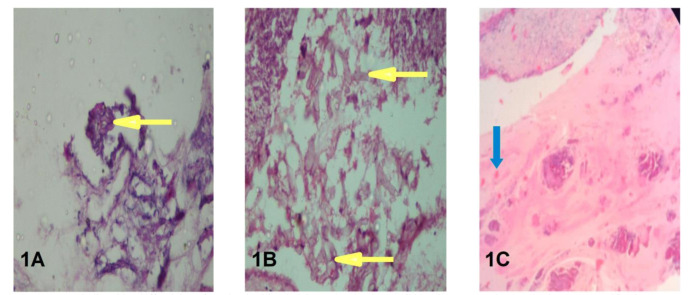
A. Periodic acid-Schiff staining of nasal polyp biopsied tissue showing broad, ribbon-like, aseptate hyphae (under 400x magnification) **Figure 1.**B. Hematoxylin and Eosin (H&E) staining of nasal polyp biopsied tissue showing fungal hyphae (under 400x magnification) **Figure 1.**C. Hematoxylin and Eosin (H&E) staining of nasal polyp biopsied tissue showing eosinophilic mucin (under 400x magnification)

Participants (n=34) in this study included 17 (50%) males and 17 (50%) females. Nasal polyp and inferior turbinate hypertrophy were the most common associated findings [Table 1]. The most common clinical presentations were nasal obstruction, nasal discharge, hyposmia/anosmia, and headache [Table 2]. All 34 cases were subjected to direct microscopic examination using KOH (20%). It was positive for fungal elements in 44.1% (15/34) of samples, which were also positive for fungal culture growth [Table 3] with additional two KOH negative and culture-positive samples. Out of all 34 samples subjected to HPE, 41.2% (14/34) were positive for both fungal elements and fungal culture growth [Figure 1, Table 3].

**Table 1:** Associated clinical findings of cases with suspected rhinosinusitis

Findings	Total cases of rhinosinusitis [%(n)] (N=34)
Fungal Polyps	100% (34)
Diabetes mellitus	11.8% (4)
Deviated nasal septum	14.7% (5)
Inferior turbinate hypertrophy	100% (34)
Use of nasal decongestants	8.8% (3)
Family history of allergy	5.9% (2)
No significant findings	0

**Table 2:** Clinical presentations in cases with suspected rhinosinusitis

Clinical presentation (signs and symptoms)	Total cases of rhinosinusitis [%(n)] (N=34)
Nasal discharge	100% (34 )
Nasal obstruction	100% (34 )
Headache	88.2% (30)
Anosmia /hyposmia	100% (34)
Allergy to dust, pollen, perfumes, etc.	29.4% (10)
Fever	29.4% (10)
Ear pain/fullness	20.6% (7)
Facial pain/Swelling	20.6% (7)
Ocular pain /proptosis	14.7% (5)

**Table 3:** Comparison of KOH mount findings and histopathological examination findings of biopsied tissue with fungal culture isolation & PCR in suspected patients of fungal rhinosinusitis

		Fungal culture and PCR of biopsied tissue (N=34)		
		Culture and PCR positive (n= 17)	Culture and PCR negative (n= 16)	Culture negative and PCR positive (n=1)
**Fungal elements on KOH mount of biopsied tissue**	Positive (n=15)	15	0	0
	Negative (n=19)	2	16	1
**Fungal elements on histopathological examination**	Positive (n=14)	14	0.	0.
	Negative (n=20)	3	16	1

A total of 50% (17/34) of samples were positive for fungal growth on culture, out of which two were negative for fungal elements on KOH mount (P<0.05), and three were negative on HPE (P<0.05) [Table 3]. Among the histological types of fungal rhinosinusitis, AFRS [41.2%; 7/17] was the most common type followed by AFIFRS [23.5%; 4/17], CIFRS [17.6%; 3/17], non-allergic FRS [11.8%; 2/17], and GIFRS [5.9%; 1/17] [Table 4]. In two out of the seven AFRS cases and one non-allergic FRS case, fungal hyphae were not identified on histopathology. Aspergillus [76.5%; 13/17] was the commonest fungal isolate found in the present study (*Aspergillus flavus* complex [58.8%; 10/17] being the most common species), followed by *Rhizopus arrhizus* [17.6%; 3/17]. Moreover, two *Aspergillus* spp. and one *Cladosporium* spp. were identified only up to the genus level.

**Table 4:** Distribution of different fungal isolates (identified on culture) from nasal biopsied tissue and radiological presentations among various histological types of fungal rhinosinusitis in suspected patients

Total fungal isolates (n=17)		AFRS (7)	Nonallergic FRS (2)	AFIFRS (4)	GIFRS (1)	CIFRS (3)
*Aspergillus flavus* complex (N=10)		6	1	1	0	2
*Aspergillus fumigatus* complex (N=1)		1	0	0	0	0
*Aspergillus spp.* (N=2)		0	0	0	1	1
*Rhizopus arrhizus* (N=3)		0	0	3	0	0
*Cladosporium spp.* (N=1)		0	1	0	0	0
CT scan findings		AFRS*	Nonallergic FRS	AFIFRS	GIFRS	CIFRS
	Intraorbital extension	0	0	2	1	0
	Bony erosion	1	0	1	1	1
	Hyperattenuation	8	2	4	1	2
Elevated IgE (N=9)		7	0	0	0	2

* It includes 7 culture-positive cases and 1 PCR-only positive case of AFRS

Abbreviations: AFRS: Allergic Fungal Rhinosinusitis, FRS: Fungal Rhinosinusitis, AFIFRS: Acute (fulminant) invasive Fungal Rhinosinusitis, GIFRS: Granulomatous Invasive Fungal Rhinosinusitis, CIFRS: Chronic Invasive Fungal Rhinosinusitis.

On computed tomography scans of paranasal sinuses, hyper-attenuation, bony erosion, and intra-orbital extension were observed in all the cases, four cases, and three cases of FRS, respectively. Serum IgE levels were found to be elevated in 9 (26.5%) out of 34 cases, out of which seven were classified as AFRS and two were classified as CIFRS.

Out of 34 samples, all 17 (100%) samples that were positive on fungal culture were also positive on pan-fungal PCR. About 94.1% (16/17) of samples that were negative on fungal culture were also negative for fungal etiology on pan-fungal PCR. However, one (5.9%) out of 17 negative samples on fungal culture was positive for fungal etiology on pan-fungal PCR and subsequently identified as *Cryptococcus heimaeyensis* by DNA sequencing of nested PCR amplicon.

## Discussion

According to the U.S. National Health Interview Survey data in 2008, RS occurred in approximately one out of every seven adults [ 17 ]. This disease affects almost 20% of the population [ 18 ]. Panda et al. [ 19 ] in their study, categorized 178 patients diagnosed with paranasal sinus mycoses into allergic (n=8), non-invasive (n=92), and invasive (n=78) disease groups, based on clinical features and radiological, surgical, histopathological, and microbiological investigations. Challa et al. [ 20 ] observed a much lower incidence of non-invasive FRS (25%) versus invasive disease (75%) with a 30% incidence of GIFRS. A study conducted by Saravanan et al. [ 21 ] from Chandigarh reported that out of 32 patients in the allergic fungal rhinosinusitis group, *Aspergillus flavus*, was the most common culture isolate found in 81% of cases, followed by *A. fumigatus* (9%), with *Bipolaris* spp. isolated in only two cases (6%). Similar to the study conducted by Saravanan et al., bony erosion in AFRS cases had also been described by Bent and Kuhn et al., with 80% of (12/15) patients having some degree of radiographic bone erosion, along with eosinophilic mucin [ 11 , 21 ]. In the present study, one AFRS case presented with minimal bony erosion. In one case of CIFRS in our study, *Cryptococcus heimaeyensis* was identified by DNA sequencing of nested PCR amplicon. This case was negative for fungal hyphae on direct microscopic examination. Chronic invasive granulomatous fungal sinusitis (CIGFS) caused by Cryptococcus is extremely rare; however, five cases of cryptococcal sinus infection have been reported so far in the literature [ 22 ]. In a study performed by Polzehl et al., fungal cultures were conducted on Nasal Lavages (NLs) from 77 patients with CRS. NLs were also tested for the presence of fungal DNA. Fungi were detected in 39 (50%) patients by the combination of culture and PCR [ 23 ]. The study results revealed that PCR and conventional culture techniques complement each other for the detection of fungi in nasal specimens from CRS patients. In the present study, FRS was diagnosed in 52.9% of RS cases (culture/PCR). In the present study, fungal culture was statistically superior to both KOH mount and histopathological staining technique, while there was no statistically significant difference between fungal culture and PCR. AFRS (41.2%) was the most common histopathological diagnosis. *A. flavus* was the most common fungus isolated from nasal specimens. Singh et al., [ 10 ] from Lucknow (Uttar Pradesh, India) reported incidence of FRS to be 48.7% with AFRS (64.2%) being the most common form of FRS based on histopathology, and A. flavus as the most common species in their study population, which confirmed the findings of the present study. Other studies, including those performed by Das et al. from Chandigarh and Prateek et al. from Lucknow, India, have reported FRS in 42.7% and 21% of cases in their respective studies [ 24 - 25 ]. Identification of fungi by molecular methods is a subset of diagnostic methods that do not necessarily need live fungal cells for success; therefore, PCR assays and sequence analysis in clinical tests for fungi facilitate early diagnosis and appropriate treatments to deal with the false negatives from culture results.

One limitation of this study is that the comparative evaluation of conventional and molecular diagnostic approaches for the diagnosis of the different clinical forms of FRS was not performed.

## Conclusion

Until now, the combination of direct microscopic examination of clinical specimens and fungal culture has been the ‘old is gold’ strategic plan, as far as mycological investigations are concerned. The molecular approaches, such as PCR, for the diagnosis of fungal rhinosinusitis, is rapid and effective tool. However, utmost precaution is needed to be taken during fungal culture and molecular detection to avoid the possibility of contamination from the environment.

## Acknowledgments

The authors would like to extend their special thanks to all the technical staff of the department of microbiology (Institute of Medical Sciences, Banaras Hindu University, Varanasi, Uttar Pradesh, India) for their co-operation and support.

## Authors’ contribution

All the authors have made a substantial, direct, and intellectual contribution to the work. 

## Conflicts of interest

None.

## Financial disclosure

The study plan was approved by the Ethics Committee of the Institute of Medical Sciences, Banaras Hindu University, Varanasi, Uttar Pradesh, India with certificate number ECR/526/Inst/UP/2014 Dt. 31/1/14, and informed consent was obtained from participants.

## References

[1] Lund VJ ( 1997). Rhinosinusitis. Br J Hosp Med.

[2] Badiee P, Gandomi B, Sabz G, Khodami B, Choopanizadeh M, Jafarian H ( 2015). Evaluation of nested PCR in diagnosis of fungal rhinosinusitis. Iran J Microbiol.

[3] Aring AM, Chan MM ( 2016). Current Concepts in Adult Acute Rhinosinusitis. Am Fam Physician.

[4] Chow AW, Benninger MS, Brook I, Brozek JL, Goldstein EJ, Hicks LA, et al ( 2012). IDSA clinical practice guideline for acute bacterial rhinosinusitis in children and adults. Clin Infect Dis.

[5] Rosenfeld RM, Piccirillo JF, Chandrasekhar SS, Brook I, Ashok Kumar K, Kramper M, Orlandi RR, Palmer JN, Patel ZM, Peters A, Walsh SA, Corrigan MD ( 2015). Clinical practice guideline (update): adult sinusitis. Otolaryngol Head Neck Surg.

[6] Hora JF ( 1965). Primary aspergillosis of the paranasal sinuses and associated areas. Laryngoscope.

[7] Jahrsdoerfer RA, Ejercito VS, Johns MM, Cantrell RW, Sydnor JB ( 1979). Aspergillosis of the nose and paranasal sinuses. Am J Otolaryngol.

[8] Lowe J, Bradley J ( 1986). Cerebral and orbital Aspergillus infection due to invasive aspergillosis of ethmoid sinus. J Clin Pathol.

[9] Chakrabarti A, Denning DW, Ferguson BJ, Ponikau J, Buzina W, Kita H, Marple B, Panda N, Vlaminck S, Kauffmann-Lacroix C, Das A, Singh P, Taj-Aldeen SJ, Kantarcioglu AS, Handa KK, Gupta A, Thungabathra M, Shivaprakash MR, Bal A, Fothergill A, Radotra BD ( 2009). Fungal rhinosinusitis: a categorization and definitional schema addressing current controversies. Laryngoscope.

[10] Singh AK, Gupta P, Verma N, Khare V, Ahamad A, Verma V, Agarwal SP ( 2017). Fungal Rhinosinusitis: Microbiological and Histopathological Perspective. J Clin Diagn Res.

[11] Bent JP 3rd, Kuhn FA ( 1994). Diagnosis of allergic fungal sinusitis. Otolaryngol Head Neck Surg.

[12] Ponikau JU, Sherris DA, Kern EB, Homburger HA, Frigas E, Gaffey TA, Roberts GD ( 1999). The diagnosis and incidence of allergic fungal sinusitis. Mayo Clin Proc.

[13] Ferguson BJ ( 2000). Eosinophilic mucin rhinosinusitis: a distinct clinicopathological entity. Laryngoscope.

[14] Chakrabarti A, Kaur H ( 2016). Allergic Aspergillus Rhinosinusitis. J Fungi (Basel).

[15] Osguthorpe JD ( 2001). Adult rhinosinusitis: diagnosis and management. Am Fam Physician.

[16] Lanza DC, Kennedy DW ( 1997). Adult rhinosinusitis defined. Otolaryngol Head Neck Surg.

[17] Pleis JR, Lucas JW, Ward BW ( 2009). Summary health statistics for U.S. adults: National Health Interview Survey, 2008. Vital Health Stat 10.

[18] Lund VJ ( 1997). Infectious rhinosinusitis in adults: classification, etiology and management. International Rhinosinusitis Advisory Board. Ear Nose Throat J.

[19] Panda NK, Sharma SC, Chakrabarti A, Mann SB ( 1998 ). Paranasal sinus mycoses in north India. Mycoses.

[20] Challa S, Uppin SG, Hanumanthu S, Panigrahi MK, Purohit AK, Sattaluri S, Borgohain R, Chava A, Vemu L, Jagarlapudi MM ( 2010). Fungal rhinosinusitis: a clinicopathological study from South India. Eur Arch Otorhinolaryngol.

[21] Saravanan K, Panda NK, Chakrabarti A, Das A, Bapuraj RJ ( 2006). Allergic fungal rhinosinusitis: an attempt to resolve the diagnostic dilemma. Arch Otolaryngol Head Neck Surg.

[22] Dai LB, Yang H, Xu B, Yong WW, Zhou SH, Bao YY, Han HM, Zhong JT, Yu E ( 2017). Primary cryptococcosis of paranasal sinus in immunocompetent patient: two case reports and review of literature. International Journal of Clinical and Experimental Medicine.

[23] Polzehl D, Weschta M, Podbielski A, Riechelmann H, Rimek D ( 2005). Fungus culture and PCR in nasal lavage samples of patients with chronic rhinosinusitis. J Med Microbiol.

[24] Das A, Bal A, Chakrabarti A, Panda N, Joshi K ( 2009). Spectrum of fungal rhinosinusitis; histopathologist's perspective. Histopathology.

[25] Prateek S, Banerjee G, Gupta P, Singh M, Goel MM, Verma V ( 2013). Fungal rhinosinusitis: a prospective study in a University hospital of Uttar Pradesh. Indian J Med Microbiol.

